# Supplementary material for: Bayesian refinement of protein structures and ensembles against SAXS data using molecular dynamics
Source: PLoS Comput Biol. 2017 Oct 18;13(10):e1005800. doi: 10.1371/journal.pcbi.1005800 (PMC5662244; doi:10.1371/journal.pcbi.1005800)
Supplement: S1 Archive — (BZ2) [file pcbi.1005800.s011.tar.bz2 › gromacs_4.62_bayesian_saxs_refi/share/html/online/getting_started.html]

Getting started


| Getting started Main Table of Contents |

**VERSION 4.6  
Sat 19 Jan 2013**

---

### Contents

- Introduction
  - Setting up your environment- Examples
  - The GROMACS demo- Water- Methanol- Water/Methanol mixture- Ribonuclease S-Peptide- Protein unfolding- GROMACS files
                - Molecular topology file- Molecular structure file- Molecular dynamics parameter file- Index file- Run input file- Trajectory file
                - References

More info can be found in the
flowchart
(for a quick overview) and the
GMX FAQ.

  

---

  


## Introduction

In this chapter we assume the reader is familiar with Molecular
Dynamics and familiar with Unix, including the use of a text editor
such as jot, emacs or vi. We furthermore assume the
GROMACS software is installed properly on your system. When you see a line
like

|  |  |  |
| --- | --- | --- |
|  | ls -l   \| |

  
you are supposed to type the contents of that line on your computer.

### Setting up your environment

In order to check whether you have access to the GROMACS software, please
start by entering the command:

|  |  |  |
| --- | --- | --- |
|  | luck   \| |

  
This command should return a cool quote like:

```
"Whatever Happened to Pong ?" (F. Black)
```

If this, in contrast, returns the phrase

```
luck: command not found.
```

then you have to verify where your version of GROMACS is installed.
In the default case, the binaries are located in
'/usr/local/gromacs/bin', however, you can ask your local system
administrator for more information. If we assume that GROMACS is
installed in directory XXX you would find the executables (programs) in
XXX/bin. To be able to access the programs without
problems, you will have to edit the login file for your shell. If you
use the C shell, this file is called .cshrc or
.tcshrc, and it is located in your home directory. Add a line
like:
  
  

|  |  |  |
| --- | --- | --- |
|  | source XXX/bin/GMXRC   \| |

  
Issue this command at the prompt too, or log off and on again to
automatically get the environment.
You should have an environment variable set now that is called
GMXDATA that we will use further on. Let us check whether this was
successful using:  
  

|  |  |  |
| --- | --- | --- |
|  | echo $GMXDATA   \| |

  
If it prints a directory name you are ready to rock, otherwise go back two steps.

### Examples

Before starting the examples, you have to copy all the neccesary
files, to your own directory, let us call it work.
Mkdir and chdir to the directory you want to put
the examples directory. This directory (named tutor)
will need
about 20 MB of disk space, when it is completely filled.  
  

|  |  |  |
| --- | --- | --- |
|  | mkdir work; cd work   \| |

  
then copy the examples:   
  

|  |  |  |
| --- | --- | --- |
|  | cp -r $GMXDATA/gromacs/tutor .   \| |

  
(NOTE: include the ".")  
If that directory doesn't exist you could look for the files
in /usr/local/share/gromacs/tutor, or ask your local GROMACS expert.
  
 You now have a subdirectory
tutor. Move there  
  

|  |  |  |
| --- | --- | --- |
|  | cd tutor   \| |

  
and view the contents of this directory
  
  

|  |  |  |
| --- | --- | --- |
|  | ls -l   \| |

  
If all is well you will have seven subdirectories with examples
with names like gmxdemo, methanol,
mixed, nmr1, nmr2,
speptide and water.

You are encouraged to look up the different programs and
file formats in 
the online manual while you are browsing through the examples.

**While going through the tutorial you will find questions
(in a red font). Try to answer them, to increase
your understanding of molecular simulation.**


### Go to the first step

  

---

  

## GROMACS files

Here is an overview of the most important GROMACS file types that you will
encounter during the tutorial.

### Molecular Topology file (.top) : The molecular topology file is generated by the program pdb2gmx. pdb2gmx translates a pdb structure file of any peptide or protein to a molecular topology file. This topology file contains a complete description of all the interactions in your peptide or protein. ### Molecular Structure file (.gro, .pdb) : When the pdb2gmx program is executed to generate a molecular topology, it also translates the structure file (.pdb file) to a gromos structure file (.gro file). The main difference between a pdb file and a gromos file is their format and that a .gro file can also hold velocities. However, if you do not need the velocities, you can also use a pdb file in all programs. To generate a box of solvent molecules around the peptide, the program genbox is used. First the program editconf should be used to define a box of appropriate size around the molecule. genbox dissolves a solute molecule (the peptide) into any solvent (in this case water). The output of genbox is a gromos structure file of the peptide dissolved in water. The genbox program also changes the molecular topology file (generated by pdb2gmx) to add solvent to the topology. ### Molecular Dynamics parameter file (.mdp) : The Molecular Dynamics Parameter (.mdp) file contains all information about the Molecular Dynamics simulation itself e.g. time-step, number of steps, temperature, pressure etc. The easiest way of handling such a file is by adapting a sample .mdp file. A sample mdp file can be found online. ### Index file (.ndx) : Sometimes you may need an index file to specify actions on groups of atoms (e.g. Temperature coupling, accelerations, freezing). Usually the default index groups will be sufficient, so for this demo we will not consider the use of index files. ### Run input file (.tpr) : The next step is to combine the molecular structure (.gro file), topology (.top file) MD-parameters (.mdp file) and (optionally) the index file (ndx) to generate a run input file (.tpr extension or .tpb if you don't have XDR). This file contains all information needed to start a simulation with GROMACS. The grompp program processes all input files and generates the run input .tpr file. ### Trajectory file (.trr) : Once the run input file is available, we can start the simulation. The program which starts the simulation is called mdrun. The only input file of mdrun you usually need to start a run is the run input file (.tpr file). The output files of mdrun are the trajectory file (.trr file or .trj if you don't have XDR) and a logfile ( .log file).

  

---

  

## References

> Berendsen, H.J.C., Postma, J.P.M., van Gunsteren, W.F., Hermans, J. (1981): Intermolecular Forces, chapter Interaction models for water in relation to protein hydration, pp 331-342. Dordrecht: D. Reidel Publishing Company Dordrecht Kabsch, W., Sander, C. (1983).: Dictionary of protein secondary structure: Pattern recognition of hydrogen-bonded and geometrical features. Biopolymers **22**, 2577--2637. Mierke, D.F., Kessler, H. (1991).: Molecular dynamics with dimethyl sulfoxide as a solvent. Conformation of a cyclic hexapeptide. J. Am. Chem. Soc. **113**, 9446. Stryer, L. (1988).: Biochemistry vol. 1, p. 211. New York: Freeman, 3 edition.

  


---

http://www.gromacs.org
